# Patient and Caregiver Perceptions of Animal Assisted Activity in Orthodontics

**DOI:** 10.3390/ani12141862

**Published:** 2022-07-21

**Authors:** Katelyn Cass, Clare Bocklage, Taylor Sulkowski, Christina Graves, Nare Ghaltakhchyan, Allen Rapolla, Tate Jackson, Kimon Divaris, Chris Wiesen, Timothy Strauman, Laura Jacox

**Affiliations:** 1 Orthodontics Group, Division of Craniofacial and Surgical Care, Adams School of Dentistry, University of North Carolina, 270 Brauer Hall, CB #270, Chapel Hill, NC 27599, USA; katie13cass@gmail.com (K.C.); eclare@live.unc.edu (C.B.); taylor.sulkowski@unc.edu (T.S.); nare@live.unc.edu (N.G.); arapolla@live.unc.edu (A.R.); tatejackson@unc.edu (T.J.); 2 Summers Orthodontics, 4207 E North St, Greenville, SC 29615, USA; 3 Division of Oral and Craniofacial Health Sciences, Adams School of Dentistry, University of North Carolina, 385 S Columbia St, CB #7455, Chapel Hill, NC 27599, USA; cxg@email.unc.edu; 4 Division of Pediatric and Public Health, Adams School of Dentistry, University of North Carolina, 385 S Columbia St, CB #7455, Chapel Hill, NC 27599, USA; kimon_divaris@unc.edu; 5 Howard W. Odum Institute for Research in Social Science, Davis Library, University of North Carolina, 208 Raleigh St, CB #3355, Chapel Hill, NC 27514, USA; chris_wiesen@unc.edu; 6 Psychology and Neuroscience Department, Trinity College of Arts and Sciences, Duke University, 417 Chapel Dr, CB #90086, Durham, NC 27514, USA; tjstraum@duke.edu

**Keywords:** dogs, dental anxiety, dental fear, anxiety, orthodontists, orthodontics, dentistry, animal therapy, animal assisted therapy, COVID-19

## Abstract

**Simple Summary:**

Dental anxiety impacts a significant fraction of children and adults, leading to lifelong avoidance of the dentist and increased emergency dental care. Animal-assisted activity (AAA) is widely used in medicine to reduce anxiety and pain, with promise in dentistry. However, dentistry has been slow to adopt AAA, with a state dental board banning therapy animals in dental clinics due to patient concerns over dog safety, allergies, and cleanliness. Our goal was to determine how orthodontic patients and their caregivers viewed canine therapists in dental clinics to see whether AAA would be welcomed by most families. (No dog therapy occurred as part of this study, so the efficacy of AAA for dental anxiety management was not evaluated). Orthodontic patients and parents/caregivers were asked to fill out a survey about their dental anxiety and their desire for and concerns regarding therapy animals in dental clinics. More than a third of patients had moderate or greater anxiety related to dental care. A vast majority of participants believed that therapy dogs would make dental experiences more enjoyable and reduce fear, with a small minority raising concerns about cleanliness, allergies, and safety. Among patients and caregivers, there is broad acceptance and desire for AAA in dental and orthodontic settings. Future research should be aimed at determining how AAA can improve the experiences of dental patients.

**Abstract:**

Dental anxiety affects up to 21% of children and 80% of adults and is associated with lifelong dental avoidance. Animal assisted activity (AAA) is widely used to reduce anxiety and pain in medical settings and has promise in dentistry. The primary objective of this study was to evaluate caregiver and patient perceptions of canine AAA in orthodontics. A cross-sectional survey consisting of pre-tested and validated questions was conducted (*n* = 800) including orthodontic patients (*n* = 352 minors, *n* = 204 adults) and parents/caregivers (*n* = 244) attending university orthodontic clinics. In this study, AAA and dog therapy were not used or tested for dental anxiety management. More than a third of orthodontic patients (37%) had moderate or greater anxiety related to care. Participants believed that therapy animals would make dental experiences more enjoyable (75%) and reduce anxiety (82%). There was little to no concern expressed regarding cleanliness (83%), allergies (81%), and safety (89%) with a therapy animal in dental settings. Almost half of the participants would preferentially select an orthodontic office offering AAA. In light of the COVID-19 pandemic, we assessed whether perceptions of AAA changed before and after the shutdown of dental offices, with no significant differences. Across patients and caregivers, the responses support the use of AAA in orthodontic settings with minimal concerns.

## 1. Introduction

Dental anxiety (DA) affects 50–80% of adults and 6–21% of children [1,2]. DA commonly emerges during childhood due to traumatic experiences and often results in lifelong distress and care avoidance [3,4]. DA presents a major challenge to optimizing oral health outcomes and is associated with increased incidence of caries, infection, and urgent care [4,5]. To care for anxious children, the American Academy of Pediatric Dentistry advocates the use of pharmacological and non-pharmacological behavior guidance techniques [6]. Pharmacological sedation, which is required for highly anxious patients, carries a low risk of respiratory depression, neurological injury, and death [7]. Due to these risks, parents may elect against the use of sedation and medications. This is especially true for orthodontics, which is elective and often delayed until patients can comply; however, this delay can cause patients to miss optimal treatment timing [8]. As a result, non-pharmacological approaches are needed for managing anxious patients in orthodontic settings.

**Figure 1:**
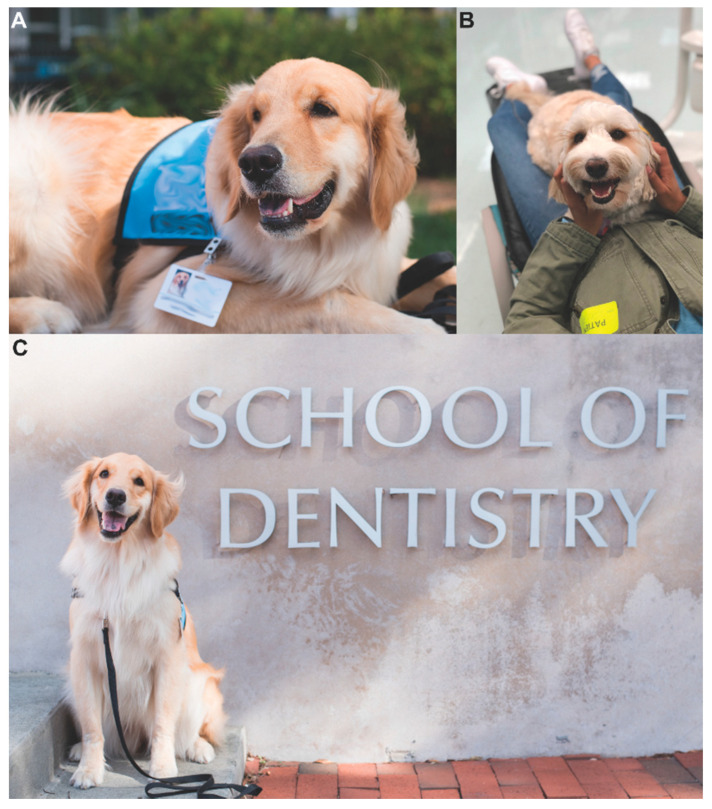
Therapy animals in dental clinics. (**A**) Certified canine therapist, Grayson Siggi. (**B**) Farley Cass comforting orthodontic patients. (**C**) Grayson welcoming visitors, as one of the first dental facility therapy dogs.

Animal assisted activity (AAA) is a promising intervention in which a certified, trained animal is introduced by a trained professional to interact with an individual to enhance their quality of life [9]. AAA is utilized to reduce anxiety, stress, and the perception of pain; it usually involves dogs that are trained to be obedient, calm, and comforting, and is an option for behavior management in dentistry (Figure 1) [10,11]. AAA distracts patients and is effective at reducing stress hormones, increasing endorphins, and activating mirror neurons [10,11,12]. AAA has been deployed successfully in inpatient and outpatient medical settings [12,13,14]. Data regarding AAA’s positive effects are abundant in medicine, however, the use of AAA in dentistry is in its nascent stages, with promising early findings [13,15,16,17,18,19]. Dental patients with a therapy animal exhibited decreased discomfort, lower blood pressure, and improvement in experience and compliance [16,17]. Among children verbalizing distress, AAA decreased their physiological arousal [17].

Though research indicates AAA’s promise for DA management, clinical adoption by practitioners depends on patient interest and acceptance [15,16,17,18]. This study aims to determine the patient perceptions of AAA in dentistry and orthodontics to inform its adoption. A review proposed that hazards of therapy animals in dental offices included safety risks, cleanliness, and allergens [20]. Safety concerns included the risk of dog bites, disease transmission (zoonosis), dog entanglement in instruments, and accidental tripping over the dog, with the potential for fall injuries [20]. Cleanliness concerns relate to waste removal (dog urine and feces) and dirt dispersion from the paws. Allergy concerns refer to airborne dander, hair shedding, and facial licking [20]. Health care protocols are used to mitigate these risks in medical and dental settings, as detailed in the guidelines for animal assisted interventions [21]. However, it is unknown whether the concerns for these hazards are held by the patients and parents/caregivers, and whether families will accept therapy animals in orthodontic clinics. To address this, we evaluated the perceptions and concerns of AAA in an orthodontic setting using pre-tested and validated survey scales. We hypothesized that AAA is acceptable to orthodontic patients and caregivers, with a majority (>70%) believing that therapy animals would make dental experiences more enjoyable, with infrequent concerns for allergies, cleanliness, and safety (<30% with medium to large concerns). Dog therapy was not performed, nor did we evaluate the efficacy of AAA for anxiety management in this study. The survey results can inform practitioners regarding AAA implementation in orthodontic contexts.

## 2. Materials and Methods

We conducted a cross-sectional survey of orthodontic patients and their parents/caregivers to determine perceptions of AAA at the University of North Carolina (UNC) Orthodontics Graduate and Faculty Clinics. Our sample (*n* = 800) included consecutively enrolled minor patients (under 18 [12–17 years old]), adult patients (>18 years old), and adult caregivers of minor patients (Table 1 and Table S1). Survey questions (File S1) were developed using pre-tested or validated questions on anxiety and AAA (data in Table 2, Table 3 and Table 4, Supplementary Tables S2–S7). The validated Corah Dental Anxiety Scale (DAS) was included as a widely used, reliable measure of anxiety; answers are scored and summed to determine anxiety level (<8 limited, 9–12 moderate, 13–14 high, 15–20 severe) (Table 4 and Table S3) [16,22,23]. Under the guidance of a survey expert, we adapted the Corah DAS to suit the orthodontic setting with minor changes, and then pre-tested and revised the questions. For topics with no published tools, the team developed, pre-tested, and revised the questions. Prior surveys and position pieces on AAA were referenced for theme inclusion [15,17,18,19]. Pre-testing was performed with iterative revisions until a final draft was approved by the investigators. Pre-testers included seven laypeople (four adults; three minors), seven residents, two private orthodontists, and two faculty. Of the pre-testers, eight owned dogs and 13 had orthodontic treatment. Among the pre-testers, individuals who did and did not own dogs were included to represent these perspectives, as dog owners were compared to non-dog owners in the survey results.

Surveys were administered using Qualtrics (Qualtrics XM, Inc., Provo, UT, USA). Potential participants were discretely approached in the clinic’s reception by study staff who screened and enrolled subjects, at 2 h intervals to ensure turnover (Table S1). Patient participants were orthodontic patients, 12–65 years old, and treated at the UNC Graduate or Faculty Orthodontics Practices (Table 1 and Table S1). Parent participants were caregivers of a minor orthodontic patient treated at the UNC Graduate or Faculty Orthodontics Practices. Study coordinators did not enroll parent–child pairs to limit any effect of their relationship on the responses. To enroll children, we would meet with the parent and child to gain parental consent and minor participant assent on IRB-approved digital forms; for these minors, we did not enroll their caregiver. To enroll caregivers, we approached them on their own, once their child was taken into the clinic for their appointment. Subjects who met the enrollment criteria (Table S1) and verbally agreed to participate were given consent forms, and then the survey to complete while they were in the orthodontics department. A screening question excluded repeat responses.

Our dental clinics host therapy dogs during clinic sessions, as part of their normal operations. A therapy dog is present during routine care in our pediatric and orthodontic clinics during most workdays; in orthodontics, the dog is seated in the reception area with her handler, or walked in the clinic by her handler to greet patients before or after their dental visit. Routine care in orthodontics includes bonding braces, removing braces, or replacing wires. Seeing a dog has the potential to influence survey participants. To control for this potential confounder and evaluate whether seeing a therapy dog influenced the perceptions of AAA, data were collected from participants with and without a therapy dog in the reception area; the dog was present on alternating weeks. The therapy dog is a 3-year-old, female, medium-sized goldendoodle who underwent therapy dog training and certification with her handler, as specified by the university with the observation of animal welfare policies (Dog: Farley Cass; Handler: Dr. Katelyn Cass, DDS, MS). During the study, the dog was seated with her handler in the reception area in an open air pen (size: 7 ft by 7 ft) positioned next to the front desk. Participants saw the dog when checking in and waiting for their appointment and could pet the dog if desired. No dog therapy occurred; we did not evaluate the efficacy of AAA for anxiety management in this study.

The survey distribution began before the COVID-19 pandemic and continued after reopening (2/2020–10/2020). Participants who took the survey prior to the shutdown (*n* = 105 minor patients, no dog) did not have questions about COVID-19. After re-opening, pandemic-related questions were added (File S1, Q29–35).

Statistics: Descriptive statistics are reported in the tables of the response frequencies. A row mean difference test was used to determine the differences between groups for ordered categorical variables. This test of equality was also used to evaluate the differences in mean scores of the outcome variable (e.g., anxiety scale value) among the grouping variable (e.g., gender, patient group). Multiple comparison tests (MCT) were conducted to evaluate the differences among groups containing three categories (e.g., under 18, over 18, and caregivers). Additionally, the Holm method was used to adjust the *p*-values of multiple comparisons to reduce the chance of type I error. McNemar’s test was used to compare dental versus orthodontic anxiety. Subjects responded to each item and the null hypothesis was that the marginal distribution of item responses was the same for both items. Matching was within subjects. Significance was defined as *p* < 0.05. Statistical analyses were conducted using SAS 9 software (SAS Institute, Cary, NC, USA). Graphs were made using Prism 9 Software (GraphPad Software Inc., La Jolla, CA, USA) and figures were created using Adobe Suite (Adobe Inc., San Jose, CA, USA).

This research was approved by the UNC Institutional Review Board (IRB #19-1908) with the protection of human subjects and their rights.

## 3. Results

### 3.1. AAA Concerns and Benefits

**Figure 2:**
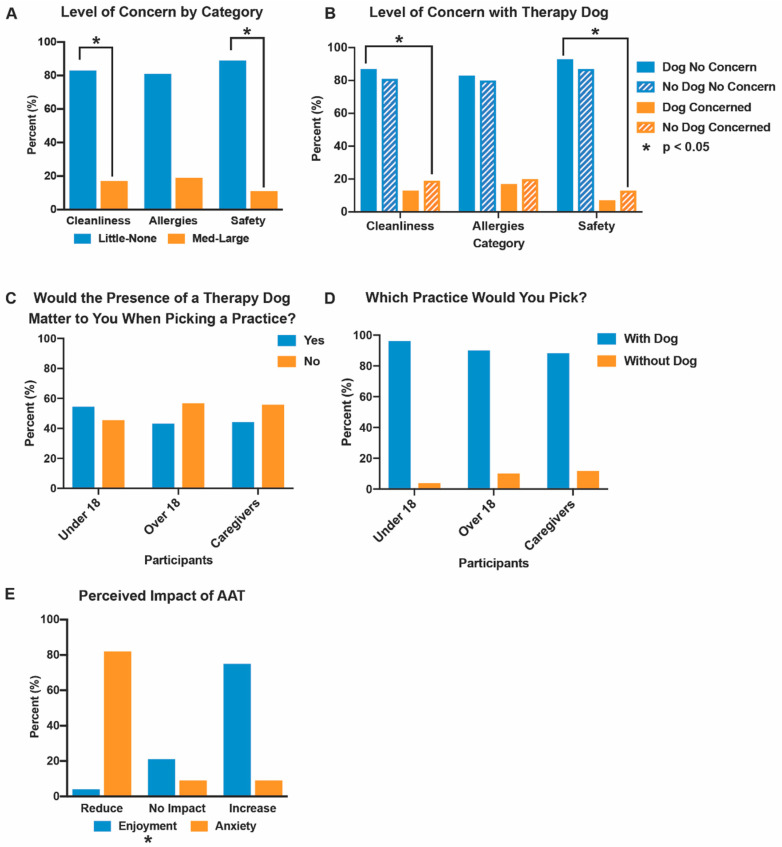
The patient and caregiver perceptions of AAA. (**A**) Frequency of participants responding “little concern” or “no concern” (pooled data, blue) versus “medium concern” and “large concern” (orange) with regard to cleanliness, allergies, and safety when having a therapy animal in a clinical dental setting. (Table 2); (**B**) Level of concern (no concern—blue; concerned—orange) about having a therapy dog present with and without a dog in the waiting area (dog present—solid; no dog present—hatched) in regard to cleanliness, allergies, and safety; (**C**) Participants responding whether the presence of a therapy dog matters (yes—blue; no—orange) to patients under 18, patients over 18, and caregivers when selecting between two similar orthodontic practices. (Table 3); (**D**) Participants (under 18 patients, over 18 patients, caregivers) responding to which practice they would pick (with a dog—blue; without a dog—orange); (**E**) Perceived impact (reduce, no impact, increase) of AAA on enjoyment (blue) and anxiety (orange) (Table S2). Statistically significant at the *p* < 0.05 level. Survey questions are in File S1.

To determine the perceptions of AAA, questions probed the concerns, desired experiences, and anxiety. The response rate was 84.9% with 800 participants including 204 adult patients, 352 minor patients, and 244 caregivers (Table 1). Data showed that a large majority of patients and caregivers reported “little” or “no concern” regarding cleanliness (83%), exposure to allergens (81%), or safety (89%) with a therapy animal in a dental setting (Figure 2A,B, Table 2). Participants with pet dogs and those who filled out the survey with a dog present more frequently reported “little” or “no concern” than participants without pet dogs and without a dog present, which is consistent with our hypotheses (Table 2).

Three quarters (75%) of participants and 85% of minors (*p* = 0.00003) indicated that having a therapy dog in a dental office would create a more enjoyable patient experience and 82% selected that the therapy animal would reduce dental anxiety (Figure 2E, Tables S2 and S8). Roughly half of the caregivers (44%) and patients (55%) under 18 indicated that the presence of a therapy animal in an orthodontic office would be important to decide which office they selected for care (Figure 2C, Table 3). Of these participants, 88% of caregivers and 96% of patients under 18 would preferentially select an office offering AAA (Figure 2D, Table 3). Dog owners were even more likely to choose an office with AAA than non-dog owners, however, 85% of those without a pet dog would still choose a practice offering AAA (Table 3). The overwhelming majority of patients and caregivers indicated that therapy animals would reduce anxiety and increase enjoyment in orthodontic settings with minimal concerns.

### 3.2. Dental Anxiety and AAA

**Figure 3:**
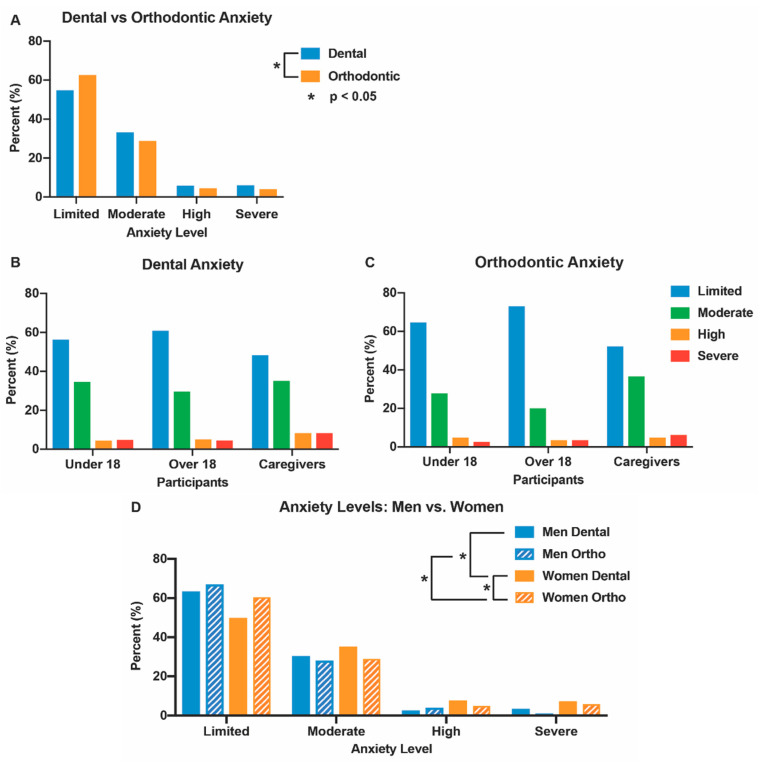
Dental and orthodontic anxiety. (**A**) Percentage of participants with limited, moderate, high, or severe anxiety (dental anxiety—blue; orthodontic anxiety—orange); (**B**) Participants (patients under 18, patients over 18, caregivers) with dental anxiety (limited—blue; moderate—green; high—orange; severe—red); (**C**) Participants (patients under 18, patients over 18, caregivers) with orthodontic anxiety (limited—blue; moderate—green; high—orange; severe—red); (**D**) Levels of dental (solid) and orthodontic (hatched) anxiety in males (blue) and females (orange). Dental and orthodontic anxiety determined by the Corah Dental Anxiety Scale (DAS) and modified orthodontic DAS, respectively (Table 4 and Table S3).

Almost half of the participants (45%) suffered from some level of anticipatory dental anxiety and 37% had orthodontic-related anxiety at a level of moderate or greater (Figure 3A, Table 4 and Table S3). Adult and minor patients reported a higher severity of dental anxiety than orthodontic anxiety (Figure 3B,C, Table 4 and Table S3). Moreover, the caregivers indicated more dental and orthodontic anxiety than adults and minor patients despite not having appointments (Figure 3B,C, Table 4, Tables S3 and S8). Females reported higher dental than orthodontic anxiety, along with more dental and orthodontic anxiety than males (Figure 3D, Table 4 and Table S3). Patients and caregivers who took the survey with a therapy dog had minimal change in the anticipated dental or orthodontic anxiety when compared to participants who did not see the dog (Table 4 and Table S3).

### 3.3. Impact of the COVID-19 Pandemic

**Figure 4:**
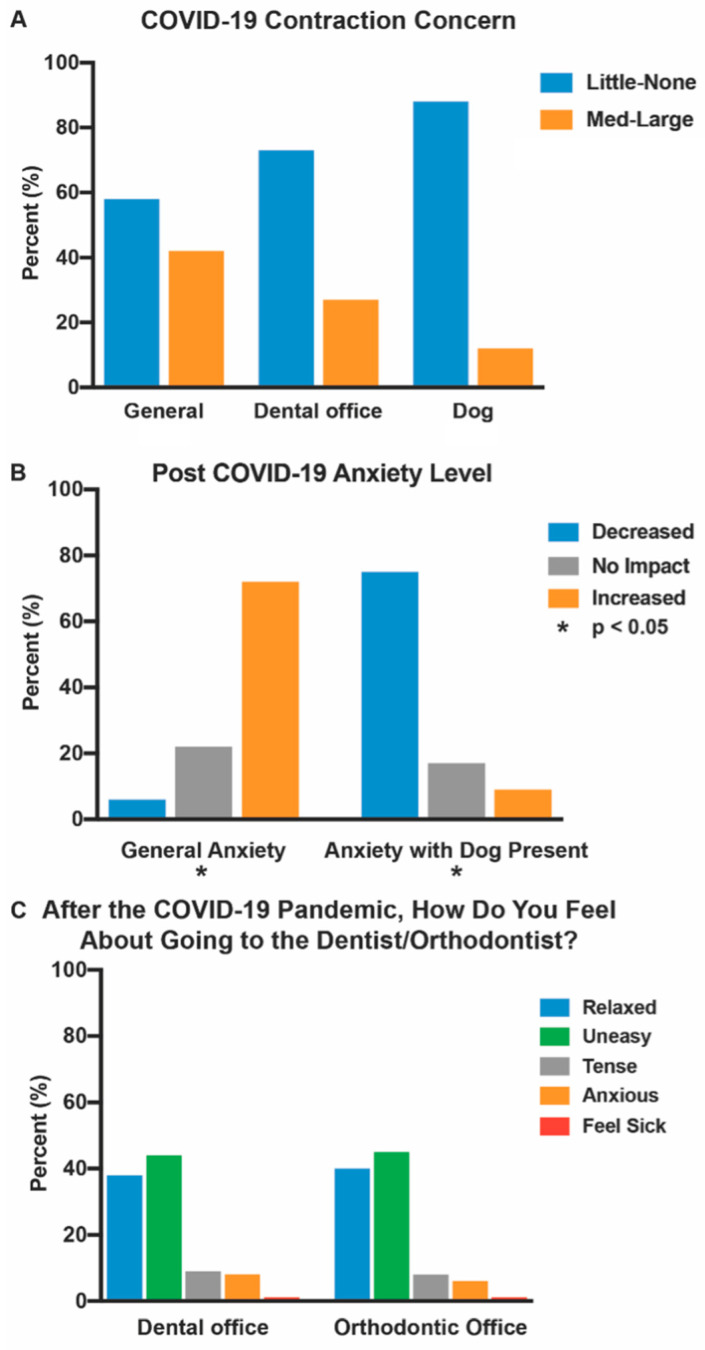
Concerns regarding COVID-19. (**A**) Percentage of participants responding “little concern” or “no concern” (pooled data, blue) versus “medium concern” and “large concern” (orange) with regard to contracting COVID-19 in general, at the dentist’s office, or from a dog (Table S4); (**B**) Perceived impact of COVID-19 on general anxiety and anxiety with a dog present (decreased- blue; no impact—grey; increase—orange) (Table S5); (**C**) Concern regarding dental professionals after COVID-19 in a dental office or orthodontic office (relaxed—blue; uneasy—green; tense—grey; anxious—orange; feel sick—red) (Table S6).

After the COVID-19 pandemic, anxiety levels increased due to pervasive uncertainty and fear of disease [24,25]. Despite reports that SARS-CoV-2 can be spread by dogs, concerns of contracting COVID-19 from a dog was low, with 88% of participants reporting “little” to “no concern” (Figure 4A, Table S4) [26,27,28,29,30,31,32,33,34,35,36]. Though most patients had little to no concern of contracting COVID-19 from dogs and the risk of zoonotic transmission is considered low, it is important for handlers and facilities with therapy animals to adhere to proper health protocols and the use of personal protective equipment, to minimize the risk of spread and ensure safety for both the canine and patients [27,28,29,30,31,32,33,34,35,36].

While 72% of participants reported an increase in general anxiety during the pandemic, with adults and caregivers having greater reported anxiety than minors, 75% of respondents indicated that their anxiety would be reduced with a therapy dog in day-to-day life (Figure 4B, Tables S5 and S8). A sizable minority (42%) reported a “moderate” to “large” concern of contracting the virus in day-to-day life while only a quarter (27%) were concerned about contracting SARS-CoV-2 at a dental or orthodontic office (Figure 4A, Table S4). Meanwhile 40% of subjects felt “relaxed” about going to an orthodontist, while a plurality of participants reported feeling “uneasy” (44%) or “tense,” “anxious” or “sick” (16%) going to a dentist or orthodontist after the outbreak (Figure 4C, Table S6). There were no differences in concern for the safety, allergies, and cleanliness of AAA, before and during the pandemic (Table S7).

## 4. Discussion

We found that most patients and caregivers would welcome AAA in orthodontics, consistent with AAA’s widespread adoption in medicine [13]. The majority of participants indicated that AAA would alleviate anxiety and offer an enjoyable experience. Furthermore, the vast majority of caregivers and patients had “little” or “no concern” regarding cleanliness, allergens, or the safety of therapy animals in dental clinics, even during the pandemic. These results are consistent with our hypothesis and the medical literature, pointing to widespread acceptance of therapy animals in diverse health care settings [19,37]. For nearly half of the participants, an office with a therapy dog would influence their choice of provider, with the vast majority (92%) choosing the practice with a dog. Taken together, our findings suggest that incorporating therapy dogs in orthodontic practice could improve the patient experience, reduce barriers to care, and provide practice growth potential.

Dog owners and participants that took the survey with a dog present were more likely to select an office offering AAA and less likely to indicate concerns about therapy dogs, suggesting dog interactions allay fears of AAA. However, the dog’s presence in the reception was unrelated to the anticipated anxiety scales for dentistry and orthodontics. This is possibly because participants had no structured interaction with the dog and did not undergo animal therapy; participants could choose to ignore the dog, and some were seated farther from the dog than others.

Across the patient groups, dental anxiety was more severe than orthodontic-related anxiety, consistent with the literature [38]. This may be due to the greater use of injections, hand pieces, and involved procedures in general dentistry [38]. Of those participants reporting orthodontic anxiety, 29% had moderate anxiety, and may benefit from anxiety-reducing interventions such as AAA. Moderate anxiety patients have stressors that can be managed in the dental clinic, while high anxiety patients require significant intervention such as anesthesia or medications [6]. Although adult patients reported lower orthodontic and dental-related anxiety than minor patients, one in five adults and one in three minors reported orthodontic-related anxiety, which is a significant fraction of patients.

When comparing across groups, minors indicated significantly higher anticipated levels of enjoyment in the presence of a therapy dog than the adults or caregivers, suggesting that the use of AAA in dental settings could be especially beneficial for pediatric patients.

Because the pandemic caused a marked increase in anxiety and depression, stress, mood disorders, and suicidal ideation, questions were added to assess the pandemic’s effects [25,39]. Most participants (72%) reported an increase in general anxiety and thought a therapy animal would reduce the day-to-day stress and dental-related anxiety, suggesting that the expanded presence of therapy animals could be beneficial in difficult times, particularly for adult patients and caregivers who reported anxiety more often compared to minors, since the pandemic [25]. Similarly, pet owners reported increases in animal engagement for emotional support during the pandemic [40]. However, the pandemic interfered with AAA delivery, with marked reductions in therapy animal visits and team availability due to paused health care programming and fewer volunteers and dogs [40]. Health care providers need to enact protocols for re-launching AAA services post-pandemic, with careful attention paid to minimize the risks posed by dogs and SARS-CoV-2 [40,41]. Though the risk of zoonotic transmission involving dogs is low, it is important for therapy dog handlers and health care facilities to enact protocols to minimize the risk of SARS-CoV-2 spread; the Centers for Disease Control (CDC) and the American Veterinary Medical Association (AVMA) have issued useful guidelines for safe practices, cleaning, and personal protective equipment usage for canine and patient safety (27–36). Specific attention needs to also be directed at designing AAA protocols for dental clinics including accommodations for children with severe dog allergies or cynophobia.

We found no differences regarding the AAA concerns of cleanliness, allergies, or safety pre- and post-pandemic, with a small proportion (12%) of participants reporting concerns of contracting SARS-CoV-2 from dogs. This finding may be related to articles regarding transmission through pets, despite the CDC stating that the risk of human–canine transmission is low [26,36,42]. Furthermore, there is no evidence that the virus can spread from the skin, fur, or hair of pets [36].

Our response rate was 84.9%, in line with similar surveys (71%–84%), suggesting that there was no undue respondent burden [23,37]. Consistent with the general population, our sample reported a 4.1% prevalence of canine allergies and 22.9% prevalence of cynophobia including 5.9% that were “somewhat” or “very” afraid of dogs (Table 1) [43].

Limitations: Participants were consecutively enrolled during a fixed time window, with no power calculation guiding sample size. However, the sample size (*n* = 800) was within a range judged as “very good” (>500) and “above the acceptable range” (300–450) for surveys [44,45]. Data on dental anxiety may have been subject to recall bias, as patients were visiting an orthodontist, and not a general dentist. There was potential for selection bias, namely volunteer bias. Data were collected at one university with its therapy dog; this sampling bias may influence generalizability to other regions and private practices. Private practices were not included due to the pandemic’s state-wide ban on non-facility dogs. Response bias may have occurred, specifically social desirability bias, whereby participants reported the desirable outcome of positive feelings toward dogs. It was infeasible for our team to re-validate the orthodontic DAS, but modifications to the DAS were pre-tested, revised, and guided by a survey expert.

Future directions include evaluating provider perspectives and enrolling patients from other regions, specialties, and private practice. Studies investigating the effects of AAA in dentistry are needed to guide protocol development and implementation. AAA has been widely adopted in medicine; dentistry and orthodontics are the next frontier due to the high prevalence of dental anxiety and AAA’s potential to mitigate stress with few perceived risks and broad patient acceptance [2,3,4,5,38].

## 5. Conclusions

- Over a third of patients under 18 have a level of orthodontic anxiety that could benefit from interventions such as AAA.
- The majority of patients and caregivers believe dental AAA will reduce anxiety and boost enjoyment.
- For nearly half of the participants, an office with a therapy dog would influence their choice of provider, with most (92%) choosing the dog.
- The majority of participants were unconcerned with the potential allergies (81%), safety risks (89%), and cleanliness (83%) of the therapy dogs.
- AAA could be a valuable practice builder and promising anxiety-management tool welcomed by most families.

## Figures and Tables

**Table 1:** Descriptive information of study participants.

Category	Group	Frequency (%) and Number (*n*) per Group *
Participant groups	Patients who are minors under 18	44.0% (*n* = 352)
	Adult patients over 18	25.5% (*n* = 204)
	Caregivers	30.5% (*n* = 244)
Race	Caucasian	68.8% (*n* = 391)
	Black	21.7% (*n* = 123)
	Asian	7.6% (*n* = 43)
	Other	1.9% (*n* = 11)
	Prefer to not answer	*n* = 232
Ethnicity	Hispanic	18.8% (*n* = 150)
	Not Hispanic	81.3% (*n* = 650)
Sex	Female	65.7% (*n* = 460)
	Male	34.3% (*n* = 240)
	Prefer to not answer	*n* = 100
Dog Allergy	Diagnosed allergy to dogs	4.1% (*n* = 30)
	No allergy	95.9% (*n* = 706)
Fear of dogs	Not at all afraid of dogs	77.1% (*n* = 566)
	Only a little afraid of dogs	17.0% (*n* = 125)
	Somewhat afraid of dogs	4.1% (*n* = 30)
	Very afraid	1.8% (*n* = 13)
Dog Presence **	Dog present **	41.0% (*n* = 328)
	No dog present **	59.0% (*n* = 472)
COVID ^	Pre-shutdown ^	16.1% (*n* = 129)
	Post-shutdown ^	83.9% (*n* = 671)
Pet at home ^^	Pet(s) at home of any species	71.0% (*n* = 512)
	No pet at home	29.0% (*n* = 288)
	Pet dog(s) ^^	60.1% (*n* = 434)
	No pet dog ^^	39.9% (*n* = 288)
		Total *n* = 800

* Participants who did not answer the questions on the demographic variables (e.g., race, gender) were not considered in the frequency calculations. ** Participants who completed the survey in the presence of a dog in the waiting room (Dog). Participants who responded to the survey without a dog in the waiting room (No dog). ^ Responses collected before the pandemic shutdown (pre-shutdown) or after the shutdown (post-shutdown). ^^ Participants with a pet dog at home (pet dog) or without a pet dog at home (no pet dog).

**Table 2:** The concerns related to animal assisted activity (AAA).

When Thinking about a Therapy Dog in a Dental Setting, How Much Concern Would You Have for Each of the Following? (Q10)						
			**Little to No Concern**	**Medium to Large Concern**	***n* ^**	*p*-Value *
Age Groups	Cleanliness	Overall	83%	17%	721 ^	0.5106
			599 ^	122 ^		
		Under 18 **	83.8%	16.2%		
			244 ^	47 ^		
		Over 18 **	80.9%	19.1%		
			157	37		
		Caregivers **	83.9%	16.1%		
			198	38		
	Allergies	Overall	81%	19%	718	0.3436
			583	135		
		Under 18	79.3%	20.7%		
			230	60		
		Over 18	84.4%	15.6%		
			162	30		
		Caregivers	80.9%	19.1%		
			191	45		
	Safety	Overall	89%	11%	718	0.9829
			642	76		
		Under 18	89.7%	10.3%		
			260	30		
		Over 18	89.1%	10.9%		
			171	21		
		Caregivers	89.4%	10.6%		
			211	25		
Dog vs. No Dog in Clinic **	Cleanliness	Dog ***	87%	13%	721	0.0213 *
			256	39		
		No Dog ***	81%	19%		
			343	83		
	Allergies	Dog	83%	17%	718	0.2877
			246	49		
		No Dog	80%	20%		
			337	86		
	Safety	Dog	93%	7%	718	0.0185 *
			274	21		
		No Dog	87%	13%		
			368	55		
Pet dog vs. No pet dog ^^	Cleanliness	Pet ^^	86.8%	13.2%	721	0.00005 *
			382	58		
		No Pet Dog ^^	77.2%	22.8%		
			217	64		
	Allergies	Pet	84.2%	15.8%	718	0.0040 *
			369	69		
		No Pet Dog	76.4%	23.6%		
			214	66		
	Safety	Pet	92.4%	7.6%	718	0.00009 *
			404	33		
		No Pet Dog	84.7%	15.3%		
			238	43		

* *p* < 0.05 statistical significance criterion. ** Orthodontic patients who were minors under 18 (Under 18). Adult orthodontic patients over 18 (Over 18). Caregivers included parents and legal guardians of orthodontic patients who were minors (Caregivers). *** Respondents who filled out the survey while a dog was in the clinic waiting room (Dog). Respondents who filled out the survey without a dog in the clinic waiting room (No Dog). ^ The number of respondents for each subgroup. ^^ Respondents with a pet dog at home (Pet dog) or without a pet dog at home (No pet dog).

**Table 3:** The AAA’s impact on patient and caregiver’s orthodontic office selection.

If You Were Making a Choice Between Two Similar Orthodontic Practices, Would the Presence of a Dog Matter to You? (Q19)				
	**Yes**	**No**	***n* ^**	***p*-Value**
Overall	48.1%	51.9%	695 ^	0.0210 *
	334 ^	361 ^		
Under 18 **	54.5%	45.5%	279	
	152	127		
Over 18 **	43.2%	56.8%	185	
	80	105		
Caregivers **	44.2%	55.8%	231	
	102	129		
Pet Dog ^^	53.8%	46.2%	422	0.0017 *
	227	195		
No Pet Dog ^^	39.2%	60.8%	273	
	107	166		
**If Answered “Yes” Above, Which Practice Would You Pick? (Q20)**				
	**With Dog**	**Without Dog**	***n* ^**	***p*-Value**
Overall	92.2%	7.8%	334 ^	0.0523 *
	308^	26^		
Under 18	96.1%	3.9%	152	
	146	6		
Over 18	90.0%	10.0%	80	
	72	8		
Caregivers	88.2%	11.8%	102	
	90	12		
Pet Dog	95.6%	4.4%	227	0.0080 *
	217	10		
No Pet Dog	85.0%	15.0%	107	
	91	16		

* *p* < 0.05 statistical significance criterion. ** Orthodontic patients who were minors under 18 (Under 18). Adult orthodontic patients over 18 (Over 18). Caregivers included the parents and legal guardians of orthodontic patients who were minors (Caregivers). ^ Number of respondents for each subgroup. ^^ Respondents with a pet dog at home (pet dog) or without a pet dog at home (no pet dog).

**Table 4:** Corah Dental and Orthodontic Modified Dental Anxiety Scales by group (Q21–28).

		Corah Dental Anxiety Category ^^					Orthodontic Anxiety Category ^^					
Group	Total *n* ^	Limited	Moderate	High	Severe	Group *p*-Value	Limited	Moderate	High	Severe	Group *p*-Value	*p*-Value Dent v. Ortho ^^
All	664 ^	364 ^	221 ^	39 ^	40 ^		416 ^	191 ^	30 ^	27 ^		0.0030 *
		54.8%	33.4%	5.9%	5.9%		62.65%	28.76%	4.52%	4.07%		
Gender						0.0001 *					0.0066 *	
Males	224	146	70	6	8		150	63	9	2		0.2360
		63.5%	30.4%	2.6%	3.5%		63.5%	30.4%	2.6%	3.5%		
Females	432	220	155	34	32		261	125	21	25		0.0001 *
		49.9%	35.2%	7.7%	7.3%		60.4%	28.9%	4.9%	5.8%		
Age group						0.0079 *					0.0007 *	
Patients Under 18	266	153	94	12	13		172	74	13	7		0.0059 *
		56.3%	34.6%	4.4%	4.8%		64.7%	27.8%	4.9%	2.6%		
Patients Over 18	174	109	53	9	8		127	35	6	6		0.0341 *
		60.9%	29.6%	5.0%	4.5%		73.0%	20.1%	3.5%	3.5%		
Caregiver	224	110	80	19	19		117	82	11	14		0.0907
		48.3%	35.1%	8.3%	8.3%		52.2%	36.6%	4.9%	6.2%		
Dog/No Dog						0.1586					0.0389 *	
Dog present	278	114	93	26	15		155	88	14	13		0.1664
		51.8%	33.5%	9.4%	5.4%		57.4%	32.6%	5.2%	4.8%		
No dog present	401	228	134	14	2		261	104	16	14		0.0007 *
		56.9%	33.4%	3.5%	6.2%		66.2%	26.1%	4.1%	3.6%		

* *p* < 0.05 statistical significance criterion. ^ Number of participants for each subgroup. ^^ Corah Dental Anxiety DAS score (dental). Orthodontic modified anxiety DAS score (ortho). Dental Anxiety Scales (DAS) are summed (four questions, 1–5 points each) to determine the anxiety level (<8 limited, 9–12 moderate, 13–14 high, and 15–20 severe).

## Data Availability

The data presented in this study are available within this article and the Supplementary Materials.

## Supplementary Materials

The following supporting information can be downloaded at: https://www.mdpi.com/article/10.3390/ani12141862/s1, Table S1: Inclusion and exclusion criteria; Table S2: AAA’s impact on anxiety and enjoyment; Table S3: Corah Dental and Orthodontic Modified Anxiety Scales Numerical; Table S4: COVID-19 concerns regarding the contraction of the virus; Table S5: The impact of COVID-19 on stress and AAA; Table S6: Feelings toward going to a dental office after the COVID-19 pandemic; Table S7: Concerns related to animal assisted activity (AAA) pre- and post-pandemic shutdown; Table S8: Multiple comparison test (MCT) results by groups; File S1: Qualtrics survey questions and text for patients and caregivers; File S2: Dental anxiety indices.
