# Supplementary material for: Patient and Caregiver Perceptions of Animal Assisted Activity in Orthodontics
Source: Animals (Basel). 2022 Jul 21;12(14):1862. doi: 10.3390/ani12141862 (PMC9312150; doi:10.3390/ani12141862)
Supplement: Supplementary file 1 [file animals-12-01862-s001.zip › FinalSupplementalFiles.pdf]

Article Title: Patient and Caregiver Perceptions of Animal Assisted Activity in Orthodontics

File S1: Qualtrics Survey Questions and Text for Patients and Caregivers

Consent Form: This research study is being conducted by the UNC Department of Orthodontics. The purpose of the study is to determine the perception of the presence of therapy animals in a dental setting. The survey will take approximately 5-10 minutes to complete. Your participation is completely voluntary, and the information you provide will be kept confidential. Results will be reported only in aggregate form. There are not any benefits of participating in the study and taking the survey. Participation will not affect orthodontic treatment in the orthodontic clinic or waiting room in any form or at any time and participation will not cost you anything. There will be approximately 600-800 people participating in this research study. We may use de-identified data and/or specimens from this study in future research without additional consent. You can withdraw from this study at any time, without penalty, and you can skip any question that you choose not to answer. There are minimal risks for participating in the survey. You may decline to participate in the study for any reason. Refusal to participate will involve no penalty or loss of benefits that you are otherwise entitled, and that you may discontinue participation at any time without penalty or loss of benefits otherwise entitled. The alternative to participating in the study is to not take the survey and to continue with normal interactions in the waiting room and graduate clinic. Please click the **arrow** on the bottom right of screen if you wish to continue.

Q0 Screening: Have you taken this survey previously?

- -Yes (1) ⊚ Conclude survey with 'Thank you' Screen
- -No (2)

Q1 Are there any pets that live at your home?

- -Yes (1)
- -No (2)

Display This Question: *If Are there any pets that live at your home? = Yes*

Q2 What kind(s) of pet(s) do you have at your home? Please select all that apply.

- -Dog (1)
- -Cat (2)
- -Bird (3)
- -Ferret (4)
- -Fish (5)
- -Turtle (6)
- -Frog (7)
- -Lizard (8)
- -Snake (9)
- -Guinea Pig (10)
- -Hamster (11)
- -Rat (12)
- -Mouse (13)
- -Rabbit (14)
- -Other: Please Specify (15)

Q3 Are you allergic to dogs?

- -Yes (1)
- -No (2)

Display This Question: *If Are you allergic to dogs? = Yes*

Q4 Have you been formally diagnosed with this allergy to dogs by a physician?

-Yes (1)

-No (2)

Display This Question: *If Are you allergic to dogs? = Yes*

Q4b What allergy symptoms do you have around dogs? Please select all that apply.

-Sneezing (1)

-Coughing (2)

-Wheezing (3)

-Rash (4)

-Hives (5)

-Anaphylaxis / difficulty breathing (6)

-Other. Please specify: (7)

Q5 How afraid of dogs would you say you are?

-Not at all afraid (1)

-Only a little afraid (2)

-Somewhat afraid (3)

-Very afraid (4)

Q6 Have you heard of therapy dogs **<u>in a dental office</u>**?

-Yes (1)

-No (2)

Display This Question: *If Have you heard of therapy dogs* ***<u>in a dental office</u>****? = Yes*

Q7 Have you seen or interacted with a therapy dog **<u>in a dental office</u>** before?

-Yes (1)

-No (2)

Q8 Have you heard about use of therapy dogs **in any other** type of health care setting? i.e therapy, counselor, hospital, nursing home, doctor's office, assisted living facility, etc.

-Yes (1)

-No (2)

Display This Question: *If Have you heard about use of therapy dogs* ***in any other*** *type of health care setting? i.e therapy, counselor, hospital, nursing home, doctor's office, assisted living facility, etc. = Yes*

Q9 Have you seen or interacted with a therapy dog **in any other** health care setting? i.e. therapy, counselor, hospital, nursing home, doctor's office, assisted living facility, etc.

-Yes (1)

-No (2)

Q10 When thinking about a therapy dog in a dental setting, **how much concern** would you have for each of the following:

	Not a concern (1)	Small Concern (2)	Medium Concern (3)	Big Concern (4)
Office Cleanliness (6)	○	○	○	○
Allergies (7)	○	○	○	○
Safety (8)	○	○	○	○

Q11 In general, how much do you think a therapy dog in dental office would **alleviate patient anxiety levels**?

-Greatly reduce (1)

-Slightly reduce (2)

-No impact (3)

-Slightly increase (4)

-Greatly increase (5)

Q12 In general, how do you think having a therapy dog would **affect your experience** in a dental office?

-A lot less enjoyable (1)

-Slightly less enjoyable (2)

-No effect (3)

-Slightly more enjoyable (4)

-A lot more enjoyable (5)

Q13 What is your age?

Q14 Which gender do you identify with?

-Male (1)

-Female (2)

-Other. Please specify: (3)

Q15 Which of the following apply to you? Please select all that apply.

-Caucasian (1)

-African American (2)

-Native American (3)

-Hispanic / Latino (4)

-Asian / Pacific Islander (5)

-Other. Please specify: (6)

Q16 Which group describes you:

-Orthodontic Patient (1)

-Parent/Guardian of Orthodontic Patient (2)

Q17 In general, how much anxiety do you have about going to the **orthodontist?**

-No anxiety at all (1)

-A little anxious (2)

-Moderately anxious (3)

-Very anxious (4)

Q18 In general, how much anxiety do you have about going to the **dentist?**

-No anxiety at all (1)

-A little anxious (2)

-Moderately anxious (3)

-Very anxious (4)

Q19 If you were making a choice between two similar orthodontic practices, **where one had a therapy dog and the other didn’t**, would this make a difference in which you chose?

-Yes (1)

-No (2)

Display This Question: *If If you were making a choice between two different* ***where one had a therapy dog and the other didn’t****, would this make a difference in which you chose? = Yes*

Q20 Which one would you pick?

-Practice with a dog (1)

-Practice without a dog (2)

We will now ask questions about your feelings when seeing a dentist.

Q21 If you had to go to the **dentist** tomorrow, how would you feel about it?

-I would look forward to it as a reasonably pleasurable experience. (1)

-I wouldn't care one way or the other. (2)

-I would be a little uneasy about it. (3)

-I would be afraid that it would be unpleasant and painful. (4)

-I would be very frightened about what the dentist might do. (5)

Q22 When you are waiting in the **dentist's** office for your turn in the chair, how do you feel?

-Relaxed (1)

-A little uneasy (2)

-Tense (3)

-Anxious (4)

-So anxious that I sometimes break out in a sweat or almost feel physically sick (5)

Q23 When you are waiting in the **dentist's** chair while s/he gets the drill ready to begin working on your teeth, how do you feel?

-Relaxed (1)

-A little uneasy (2)

-Tense (3)

-Anxious (4)

-So anxious that I sometimes break out in a sweat or almost feel physically sick (5)

Q24 You are in a **dentist's** chair to get your teeth cleaned. While you are waiting and the dentist is getting out the instruments which s/he will use to scrape your teeth around your gums, how do you feel?

-Relaxed (1)

-A little uneasy (2)

-Tense (3)

-Anxious (4)

-So anxious that I sometimes break out in a sweat or almost feel physically sick (5)

Q25 If you had to go to the **orthodontist** tomorrow, how would you feel about it?

-I would look forward to it as a reasonably pleasurable experience. (1)

-I wouldn't care one way or the other. (2)

-I would be a little uneasy about it. (3)

-I would be afraid that it would be unpleasant and painful. (4)

-I would be very frightened about what the orthodontist might do. (5)

Q26 When you are waiting in the **orthodontist's** office for your turn in the chair, how do you feel?

-Relaxed (1)

-A little uneasy (2)

-Tense (3)

-Anxious (4)

-So anxious that I sometimes break out in a sweat or almost feel physically sick (5)

Q27 When you are waiting in an **orthodontist's** chair while s/he gets ready to begin putting your braces on, how do you feel?

-Relaxed (1)

-A little uneasy (2)

-Tense (3)

-Anxious (4)

-So anxious that I sometimes break out in a sweat or almost feel physically sick (5)

Q28 You are in an **<u>orthodontist's</u>** chair to get your braces tightened. While you are waiting and the orthodontist is getting out the instruments which s/he will use to tighten your braces, how do you feel?

-Relaxed (1)

-A little uneasy (2)

-Tense (3)

-Anxious (4)

-So anxious that I sometimes break out in a sweat or almost feel physically sick (5)

Q29 After the outbreak of COVID-19, how has your **day to day** stress or anxiety levels changed?

-My stress/anxiety has increased a lot (2)

-My stress/anxiety has increased a little (3)

-My stress/anxiety has not changed (4)

-My stress/anxiety has decreased a little (5)

-My stress/anxiety has decreased a lot (6)

Q30 After the outbreak of COVID-19, how do you feel about **going to a dental office**?

-Relaxed (1)

-A little uneasy (2)

-Tense (4)

-Anxious (5)

-So anxious that I sometimes break out in a sweat or almost feel physically sick (6)

Q31 After the outbreak of COVID-19, how do you feel about **going to the orthodontist**?

-Relaxed (1)

-A little uneasy (2)

-Tense (4)

-Anxious (5)

-So anxious that I sometimes break out in a sweat or almost feel physically sick (6)

Q32 After the outbreak of COVID-19, how concerned are you about **contracting the virus**?

-Not concerned (1)

-Small concern (2)

-Medium concern (4)

-Large concern (5)

Q33 After the outbreak of COVID-19, how concerned are you about contracting the virus **in a dental or orthodontic office**?

-Not concerned (1)

-Small concern (2)

-Medium concern (7)

-Large concern (8)

-So concerned I considered canceling my appointment (4)

Q34 In general, how much do you think a therapy dog in dental office would **alleviate patient anxiety levels** related to COVID-19?

-Greatly reduce (1)

-Slightly reduce (2)

-No impact (3)

-Slightly increase (4)

-Greatly increase (5)

Q35 When thinking about a therapy dog in a dental setting, **how much concern** would you have regarding the therapy animal as a possible vector or pathway to transmit COVID-19?

-Not concerned (1)

-Small concern (2)

-Medium concern (3)

-Large concern (4)

File S2: Dental Anxiety Indexes

<u>CORAH Dental Anxiety Scale (DAS)</u>

1.) If you had to go to the dentist tomorrow for a check-up, how would you feel about it?

A. I would look forward to it as a reasonably enjoyable experience
B. I wouldn't care one way or the other.
C. I would be a little uneasy about it.
D. I would be afraid that it would be unpleasant and painful.
E. I would be very frightened of what the dentist would do.

2.) When you are waiting in the dentist's office for your turn in the chair, how do you feel?

A. Relaxed.
B. A little uneasy
C. Tense
D. Anxious
E. So anxious that I sometimes break out in a sweat or almost feel physically sick.

3.) When you are in the dentist's chair waiting while the dentist gets the drill ready to begin working on your teeth, how do you feel?
(Same answers as number 2)

4.) Imagine you are in the dentist's chair to have your teeth cleaned. While you are waiting and the dentist or hygienist is getting out the instruments, which will be used to scrape your teeth around the gums, how do you feel?
(Same answers as number 2)

<u>Scoring:</u>

Points are assigned for a subject's choices, with one point for (a) choice to 5 points for an (e) choice (a=1; b=2; c=3; d=4; e=5; Total possible score=20)
8 or less = limited anxiety
9-12 = moderate anxiety but have specific stressors that should be discussed and managed
13-14 = high anxiety
15-20 = severe anxiety or phobia. May be manageable with the Dental Concerns Assessment but might require the help of a mental health therapist.

<u>Modified Orthodontic CORAH DAS</u>

1.) If you had to go to the orthodontist tomorrow for a check-up, how would you feel about it?

A. I would look forward to it as a reasonably enjoyable experience
B. I wouldn't care one way or the other.
C. I would be a little uneasy about it.
D. I would be afraid that it would be unpleasant and painful.
E. I would be very frightened of what the dentist would do.

2.) When you are waiting in the orthodontist's office for your turn in the chair, how do you feel?

A. Relaxed.
B. A little uneasy
C. Tense
D. Anxious
E. So anxious that I sometimes break out in a sweat or almost feel physically sick.

3.) When you are in the orthodontist's chair waiting while the orthodontist gets ready to begin putting braces on your teeth, how do you feel?
(Same answers as number 2)

4.) Imagine you are in the orthodontist's chair to get your braces tightened. While you are waiting and the orthodontist is getting out the instruments which will be used to tighten your braces, how do you feel?
(Same answers as number 2)

Scoring:
Points are assigned for a subject's choices, with one point for (a) choice to 5 points for an (e) choice (a=1; b=2; c=3; d=4; e=5; Total possible score=20)
8 or less = limited anxiety
9-12 = moderate anxiety but have specific stressors that should be discussed and managed
13-14 = high anxiety
15-20 = severe anxiety or phobia. May be manageable with the Dental Concerns Assessment but might require the help of a mental health therapist.
