# Supplementary material for: Patient and Caregiver Perceptions of Animal Assisted Activity in Orthodontics
Source: Animals (Basel). 2022 Jul 21;12(14):1862. doi: 10.3390/ani12141862 (PMC9312150; doi:10.3390/ani12141862)
Supplement: Supplementary file 1 [file animals-12-01862-s001.zip › FinalSupplementalTables.pdf]

Article Title: Patient and Caregiver Perceptions of Animal Assisted Activity in Orthodontics

Supplemental Tables

Table S1: Inclusion and Exclusion Criteria

Inclusion Criteria *	Exclusion Criteria
-Orthodontic patient in the UNC Orthodontics Graduate or Faculty practice (12-17yo or 18+yo)<br>OR<br>-Parent or caregiver of a minor patient in the UNC Orthodontics Graduate or Faculty Practice	-Under the age of 12yo<br>-Prior participant in the survey<br>-Non-fluent English speaker or reader (cannot read English)<br>-Does not consent to participate<br>-For patients- Not undergoing orthodontic care<br>-For caregivers- children not undergoing orthodontic care<br>-Only one caregiver per family unit could be included

* Participant groups include: orthodontic patients who are minors under 18 (Under 18); adult orthodontic patients over 18 (Over 18); and caregivers including parents and legal guardians of orthodontic patients who are minors (Caregivers).

Table S2: AAA's Impact on Anxiety and Enjoyment

In general, how much do you think a therapy dog in a dental office would alleviate patient anxiety levels (Q11) or affect your experience (Q12)?						
		Reduce	No Impact	Increase	*n*	*p*-Value
Anxiety (Q11)	*Overall*	81.9%<br>585 ^	9.3%<br>66 ^	8.8%<br>63 ^	714 ^	0.850
	Under 18 **	82.5%<br>236	6.7%<br>19	10.8%<br>31	714	0.816
	Over 18 **	81.3%<br>156	11.5%<br>22	7.2%<br>14		
	Caregivers **	81.8%<br>193	10.6%<br>25	7.6%<br>18		
	Pet dog ^^	88.0%<br>381	6.2%<br>27	5.8%<br>25	433	4.85E-08 *
	No pet dog ^^	72.6%<br>204	13.9%<br>39	13.5%<br>38	281	
Enjoyment (Q12)	*Overall*	4.2%<br>30	20.7%<br>147	75.1%<br>534	711	0.00003 *
	Under 18	1.0%<br>3	13.6%<br>39	85.4%<br>244	711	0.000001*
	Over 18	5.2%<br>10	27.6%<br>53	67.2%<br>129		
	Caregivers	7.3%<br>17	23.6%<br>55	69.1%<br>161		
	Pet dog	1.9%<br>8	16.4%<br>71	81.7%<br>353	432	1.97E-09 *
	No pet Dog	7.9%<br>22	27.2%<br>76	64.9%<br>181	279	

* $p < 0.05$ statistical significance criterion.
** Orthodontic patients who are minors under 18 (Under 18). Adult orthodontic patients over 18 (Over 18). Caregivers include parents and legal guardians of orthodontic patients who are minors (Caregivers).
^ Number of participants for each subgroup
^^ Participants with a pet dog at home (pet dog) or without a pet dog at home (no pet dog)

Table S3: Corah Dental and Orthodontic Modified Anxiety Scales Numerical

		Corah Dental DAS^^				Orthodontic Modified DAS^ ^			
Group	*n* ^	Mean +/- SE	Median	Range	*p*-value *	Mean +/- SE	Median	Range	*p*-value *
All	664 ^	8.49 +/- 0.13 **	8	4-20		7.76+/-0.12 **	7	4-20	
Gender					<0.0001 *				0.0003 *
Male	224	7.77+/- 0.19	7	4-19		7.16+/-0.18	6.5	4-17	
Female	432	8.89 +/- 0.17	9	4-20		8.09+/-0.16	7	4-20	
Age group					0.0324 *				0.0012 *
Under 18	266	8.26+/-0.20	8	4-20		7.56+/-0.18	7	4-17	
Over 18	174	8.24+/-0.23	8	4-18		7.29+/-0.22	7	4-17	
Caregivers	224	8.96+/-0.24	9	4-20		8.36+/-0.22	8	4-20	
Dog/No Dog					0.1300				0.0281 *
Dog present	278	8.73+/-0.20	8	4-20		8.08+/-0.20	7	4-20	
No dog present	401	8.33+/-0.17	8	4-19		7.54+/-0.15	7	4-20	

* $p < 0.05$ statistical significance criterion.
** Mean DAS score for each group, plus or minus standard error. Dental Anxiety Scales (DAS) are summed (4 questions, 1-5 points each) to determine anxiety level (< 8 limited, 9-12 moderate, 13-14 high, and 15-20 severe).
^ Number of participants for each subgroup
^^ Dental anxiety DAS score (dental). Orthodontic modified anxiety DAS score (orthodontic).

Table S4: COVID-19 concerns regarding contraction of virus

After the outbreak of COVID-19, how concerned are you about contracting the virus in general (Q32), in a dental or orthodontic office (Q33), or from a dog as a vector for transmission (Q35)?					
		Little to No Concern	Med to Large Concern	*n* ^	*p*-Value
Contraction of Virus in General (Q32)	*Overall*	57.7%<br>326 ^	42.3%<br>239 ^	565 ^	0.0794
	Under 18 **	64%<br>106	36%<br>60	166	
	Over 18 **	52%<br>89	48%<br>83	172	
	Caregivers **	58%<br>131	42%<br>96	227	
Contraction of a virus in a Dental or Orthodontic office (Q33)	*Overall*	73%<br>410	27%<br>152	562	0.9466
	Under 18	75%<br>123	25%<br>41	164	
	Over 18	71%<br>121	29%<br>50	171	
	Caregivers	73%<br>166	27%<br>61	227	
Contraction of the virus from a dog (Q35)	*Overall*	88.2%<br>493	11.8%<br>66	559	0.4122
	Under 18	91%<br>146	9%<br>15	161	
	Over 18	86%<br>147	14%<br>24	171	
	Caregivers	88%<br>200	12%<br>27	227	

* $p < 0.05$ statistical significance criterion.
** Orthodontic patients who are minors under 18 (Under 18). Adult orthodontic patients over 18 (Over 18). Caregivers include parents and legal guardians of orthodontic patients who are minors (Caregivers).
^ Number of participants for each subgroup

Table S5: Impact of COVID-19 on stress and AAT

After the outbreak of COVID-19, how has your day-to-day stress changed? (Q29) How do you think a therapy dog in a dental office would change anxiety levels related to COVID-19? (Q34)						
		Increase	No Impact	Reduce	*n* ^	*p*-Value
Day-to-Day Stress (Q29)	*Overall*	72.2%<br>410 ^	22.0%<br>125 ^	5.8%<br>33 ^	568 ^	0.00006 *
	Under 18 **	57.7%<br>97	32.8%<br>55	9.5%<br>16	168	
	Over 18 **	79.8%<br>138	14.4%<br>25	5.8%<br>10	173	
	Caregivers **	77.1%<br>175	19.8%<br>45	3.1%<br>7	227	
Therapy Dog's Effect on Stress (Q34)	*Overall*	8.6%<br>48	16.8%<br>94	74.6%<br>417	559	0.3675
	Under 18	8.0%<br>13	14.1%<br>23	77.9%<br>127	163	
	Over 18	7.1%<br>12	17.6%<br>30	75.3%<br>128	170	
	Caregivers	10.2%<br>23	18.1%<br>41	71.7%<br>162	226	

* $p < 0.05$ statistical significance criterion.
** Orthodontic patients who are minors under 18 (Under 18). Adult orthodontic patients over 18 (Over 18). Caregivers include parents and legal guardians of orthodontic patients who are minors (Caregivers).
^ Number of participants for each subgroup

Table S6: Feelings towards going to a dental office after the COVID-19 pandemic

After the outbreak of COVID-19, how do you feel about going to the dentist (Q37) or orthodontist (Q38)?							
	Relaxed	Uneasy	Tense	Anxious	Feel Sick	*n* ^	*p*-Value
Dentist (Q37)	38.2%<br>216 ^	44.4%<br>251 ^	8.7%<br>49 ^	7.6%<br>43 ^	1.1%<br>6 ^	565 ^	0.1735
Orthodontist (Q38)	40.3%<br>227	44.9%<br>253	8.1%<br>45	6.2%<br>35	0.5%<br>3	563	

* $p < 0.05$ statistical significance criterion.
^ Number of participants for each subgroup

Table S7: Concerns related to Animal Assisted Activity (AAA) Pre- and Post-Pandemic Shutdown

When thinking about a therapy dog in a dental setting, how much concern would you have for each of the following? (Q10)					
		Little to No Concern	Medium to Large Concern	*n* ^	*p*-Value
Cleanliness	Pre-shutdown	85.0%<br>91 ^	15.0%<br>16 ^	107 ^	0.201
	Post-shutdown	83.2%<br>153	16.8%<br>31	184	
Allergies	Pre-shutdown	75.5%<br>80	24.5%<br>26	106	0.245
	Post-shutdown	81.5%<br>150	18.5%<br>34	184	
Safety	Pre-shutdown	88.7%<br>94	11.3%<br>12	106	0.300
	Post-shutdown	90.2%<br>166	9.8%<br>18	184	

* $p < 0.05$ statistical significance criterion.
^ Number of participants for each subgroup. The responses collected before the pandemic shutdown were from patients under the age of 18. Therefore, comparisons are between minor patients before (Pre-shutdown) and after the pandemic shutdown (Post-shutdown).

Table S8: Multiple Comparison Test (MCT) Results by Group

	Pairs		*p*-Value *
If you were making a choice between two similar orthodontic practices, would the presence of a dog matter to you? (Q19)	Under 18 **	Over 18 **	0.0537
	Under 18	Caregivers **	0.0537
	Over 18	Caregivers	0.8523
If answered "yes" above, which practice would you pick? (Q20)	Under 18	Over 18	0.1327
	Under 18	Caregivers	0.0526
	Over 18	Caregivers	0.7063
Corah Dental Anxiety Category (Q21-24)	Under 18	Over 18	0.5398
	Under 18	Caregivers	0.0265 *
	Over 18	Caregivers	0.0197 *
Orthodontic Anxiety Category (Q25-28)	Under 18	Over 18	0.2423
	Under 18	Caregivers	0.0104 *
	Over 18	Caregivers	0.0017 *
In general, how much do you think a therapy dog in a dental office would affect your experience (Q12)?	Under 18	Over 18	<0.0001 *
	Under 18	Caregivers	<0.0001 *
	Over 18	Caregivers	0.9760
After the outbreak of COVID-19, how concerned are you about contracting the virus in general (Q32)?	Under 18	Over 18	0.0734
	Under 18	Caregivers	0.4385
	Over 18	Caregivers	0.4385
After the outbreak of COVID-19, how concerned are you about contracting the virus from a dog as a vector for transmission (Q35)?	Under 18	Over 18	0.5482
	Under 18	Caregivers	0.8427
	Over 18	Caregivers	0.8427
After the outbreak of COVID-19, how has your day-to-day stress changed? (Q29)	Under 18	Over 18	0.0003 *
	Under 18	Caregivers	<0.0001 *
	Over 18	Caregivers	0.9969
How do you think a therapy dog in a dental office would change anxiety levels related to COVID-19? (Q34)	Under 18	Over 18	0.7970
	Under 18	Caregivers	0.6055
	Over 18	Caregivers	0.6055
Corah Dental DAS	Under 18	Over 18	0.0593
	Under 18	Caregivers	0.9391
	Over 18	Caregivers	0.0584
Orthodontic Modified DAS	Under 18	Over 18	0.0019 *
	Under 18	Caregivers	0.3594
	Over 18	Caregivers	0.0092 *

* $p < 0.05$ statistical significance criterion. Pair-wise test.
** Orthodontic patients who are minors under 18 (Under 18). Adult orthodontic patients over 18 (Over 18). Caregivers include parents and legal guardians of orthodontic patients who are minors (Caregivers).
